# Using implementation science frameworks to translate and adapt a pregnancy app for an emerging Latino community

**DOI:** 10.1186/s12905-022-01975-9

**Published:** 2022-09-21

**Authors:** Anabel F. Castillo, Alexander L. Davis, Tamar Krishnamurti

**Affiliations:** 1 Naima Health LLC, 4615 Forbes Avenue, Suite 400, Pittsburgh, PA 15213 USA; 2 Department of Engineering and Public Policy, Carnegie Mellon University, Pittsburgh, PA USA; 3 Division of General Internal Medicine, Center for Innovative Research on Gender Health Equity (CONVERGE), University of Pittsburgh, Pittsburgh, PA USA

**Keywords:** Pregnancy, mHealth, Mobile application, App, Formative evaluation, Implementation science, Health disparities, Latina, Latino, Emerging communities, Culturally sensitive, Translation

## Abstract

**Background:**

Digital mobile health (mHealth) applications are a popular form of prenatal education and care delivery in the U.S.; yet there are few Spanish language options for native speakers. Furthermore, existing applications do not consider cultural differences and disparities in healthcare access, including those specific to emerging Latino communities.

**Objective:**

To adapt and translate an English-language pregnancy mobile health app to meet the language and cultural needs of Spanish-speaking Latino immigrants living in the United States.

**Methods:**

We use a multi-step process, grounded in implementation science frameworks, to adapt and translate the contents of an existing pregnancy app. Interviews with stakeholders (n = 12) who advocate for the needs of pregnant individuals in an emerging Latino community were used to identify domains of possible disparities in access to prenatal care. We then conducted semi-structured interviews with peripartum Spanish-speaking Latino users (n = 14) to understand their perspectives within those domains. We identified a list of topics to create educational material for the modified app and implemented a systematic translation approach to ensure that the new version was acceptable for immigrants from different countries in Latin America.

**Results:**

The interviews with stakeholders revealed seven critical domains that need to be addressed in an adapted prenatal app: language and communication, financial concerns, social support, immigration status, cultural differences, healthcare navigation, and connection to population-specific community resources that offer Spanish language services. The interviews with peripartum Spanish-speaking Latino women informed how the existing content in the app could be adjusted or built upon to address these issues, including providing information on accessing care offered in their native language and community support. Finally, we used a systematic approach to translate the existing application and create new content.

**Conclusion:**

This work illustrates a process to adapt an mHealth pregnancy app to the needs of an emerging Latino community, by incorporating culturally sensitive Spanish language content while focusing on addressing existing health disparities.

## Background

Of annual U.S. births, 23% are to immigrants [1]. As a result, access and delivery of high-quality healthcare for immigrant maternal populations, particularly non-English speakers or those with limited fluency, is an essential public health issue [2]. Specifically, the American College of Physicians calls for measures, such as supporting safety-net healthcare facilities and eliminating restrictions based on immigration status, to address potential disparities in care for immigrant populations proactively. This call necessitates that healthcare delivery through technology, including mobile health (mHealth) apps, also addresses the needs of diverse people [3] by considering the existing disparities associated with migration status and financial instability.

The prenatal period is particularly vulnerable for non-English speaking immigrants due to disparities in care and experience, ranging from stressors related to immigration (including lack of stable immigrant status), language difficulties, distance to care, systemic marginalization, and stigma [4, 5]. This is even more pronounced in immigrant communities that are rapidly growing with proportionally smaller Latino populations, often referred to as emerging Latino communities [6]. Immigrants in emerging Latino communities face further challenges due to a lack of local services in their native language and social support [7]. Latino immigrants settling in nontraditional destinations usually do not have the strong social support networks that other large, well-established Latino communities offer [8].

Half of the total births to U.S. immigrants are of Hispanic or Latino origin. However, Spanish-speaking Latino individuals are often at a disadvantage when using mHealth pregnancy apps due to the lack of culturally sensitive and language-specific design [9–12]. In general, even with the widespread use of smartphones, mHealth usage patterns vary widely by race, ethnicity, and English proficiency [13, 14]. mHealth pregnancy apps have the potential to offer personalized communication directly to pregnant people and identify pregnancy-related health issues earlier than may be possible with routine prenatal care [15, 16]. Nevertheless, the benefit of existing prenatal apps may be limited for immigrant populations if they fail to address users' needs in the context of existing health and cultural disparities [17]. A one-size-fits-all approach to app design may benefit the health outcomes of majority populations while sustaining, or perhaps even creating, new health disparities among non-white individuals, particularly those from Black and Hispanic communities [18]. Previous studies of mHealth pregnancy apps usually exclude non-English speakers [11], as very few pregnancy apps are multilingual. Moreover, usability and feasibility studies of mHealth pregnancy apps have generally excluded the perspective of Spanish-speaking Latino pregnant individuals in the U.S. [19].

MyHealthyPregnancy™ (MHP) was developed as a provider-prescribed app to aid risk assessment and communication between pregnant individuals and their providers. This patient-facing mobile health app and accompanying provider-facing information portal was developed with a user-centered design approach to serve the needs of individuals with high-risk pregnancies [20]. In a proof-of-concept study, Krishnamurti et al. found high app engagement levels among recruited patients, with the most consistent use among individuals from historically under-resourced communities and those with pregnancy risk factors. This app has subsequently been shown to be effective at identifying those experiencing both psychosocial and clinical risks associated with maternal mortality [16, 21].

MHP was designed to be used within a health care system. A provider recommends the MHP app to adult pregnant patients at their first prenatal appointment. Data entered into the app then tailors the user experience, including the educational content offered. The app also offers relevant resources (e.g., connection to local health services) or actions (e.g., prompts to call the prenatal care practitioner). Additionally, with patient consent, select data on specific risk factors identified through the app (e.g., depression or reports of decreased fetal movement) are securely transferred to the portal that practitioners can access. All content found in the app was developed with and reviewed by a clinical education team.

Here, we apply methods grounded in established implementation science frameworks, to translate and modify the content of the MyHealthyPregnancy app to address pregnant Spanish-speaking Latino individuals' cultural context and linguistic needs. Herein, we use the term ‘Spanish-speaking Latino’ individuals as a shorthand to refer to Latino pregnant people who speak only Spanish or have limited fluency in English.

## Methods

To adequately modify our existing pregnancy app to address domains of disparity common to Spanish-speaking Latino people in a specific emerging Latino community (Pittsburgh, PA, USA), we drew from the Transcreation Framework developed by Nápoles et al. [22]. Transcreation outlines the steps for a community-engaged process of planning, delivering, and evaluating interventions to reduce health disparities in specific underserved communities. The goal of this framework is to create health interventions that can improve health outcomes and are acceptable to the specific communities they aim to serve.

There are seven steps described by the Transcreation Framework [22]: 1) identify community infrastructure and engage partners, 2) specify theory, 3) identify multiple inputs for the new program, 4) design intervention prototype, 5) design study, methods, and measures for community setting, 6) build community capacity for delivery, and 7) deliver the intervention. In the scope of this paper, we outline the specific steps taken to design a prototype that addresses current disparities in healthcare access for an emerging Latino community. To do this, we focus on the first four steps of the Transcreation Framework. The first three allow us to identify and understand disparities in healthcare access. In the fourth step, we use the knowledge acquired from the first three steps to create a list of requirements for re-designing our app.

For the first step, we identified existing stakeholders in the community and discussed local disparities in care access for pregnant Spanish-speaking Latino individuals. For the second step, we identified a theory by mapping out literature about existing prenatal care and general health disparities that exist in Spanish-speaking Latino communities, using a methodology based on the models created by Woodward et al*.* [24] Our third step used input from qualitative interviews with community members to identify how to modify existing app content to address their needs. Finally, in step four we outlined the requirements for the app prototype, including a systematic translation approach to address language limitations that may manifest when using conventional translation methods.

### Step 1: Identify community infrastructure and engage partners

Our initial step was to identify current infrastructure and partners with relevant experience to understand the context in which the app would be delivered. For this purpose, we engaged several stakeholders who treat, teach and support Spanish-speaking Latino individuals. We began with community outreach and word-of-mouth to identify stakeholders in Pittsburgh, PA, which is currently considered a U.S. emerging Latino community [23]. Our focus was on healthcare and community organizations and educators who serve Spanish-speaking Latino patients. These stakeholders then recommended contacts they believed could offer additional insight (e.g., local Spanish-speaking doulas). Included in these informational interviews were three healthcare providers, three prenatal educators, two doulas, two leaders from community organizations, one academic researcher, and one social worker.

### Step 2: Specify a theory

In this step, we reviewed published literature to determine content areas in the app, which could be built out to address health disparities specific to this population. The Health Equity Implementation Framework (HEIF) by Woodward et al. [24] was used to organize our published literature search around the different components believed to predict the successful and equitable implementation of new health interventions.

A review of the literature in these areas identified eight primary domains of disparity for the pregnant Spanish-speaking Latino population: language and communication (limited English proficiency), language of service provision, financial concerns, immigration status, cultural factors, location of services, healthcare navigation, and health literacy [23, 25–32]. In addition to these contextual factors, Woodward et al. also place a focus on the clinical encounter. The quality of the patient-provider relationship within a clinical encounter can directly affect health care access disparities. Specifically, the quality of the patient-provider relationship may affect which interventions a provider offers to a patient and the manner by which those are offered and received. If there is trust, for example, in the patient-provider relationship, a patient may be more likely to accept and use a prescribed intervention (or the reverse, if mutual trust is lacking). In the instance of our intervention, the use of a culturally sensitive mHealth pregnancy app may support communication between patient and provider by incorporating topical content that might have been missed during the encounter or offering the language needed by the patient to clarify open questions with the provider.

**Fig. 1:**
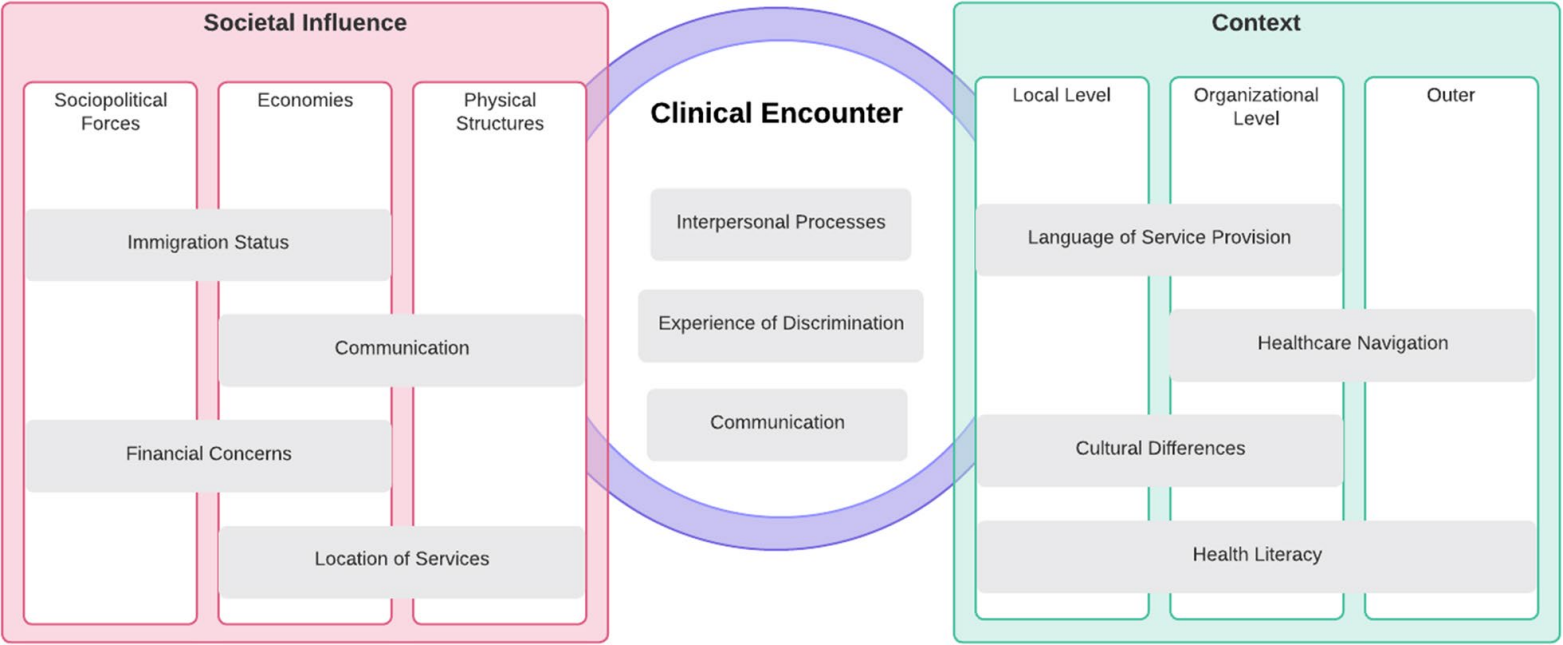
Implementation and health equity determinants adapted from Woodward et al. [24]

Figure 1 illustrates how the 8 domains identified in the literature and interacting with the clinical encounter, map to the Health Equity Implementation Framework. Together, these domains reveal ways in which societal influence and context may affect engagement with healthcare interventions, including mobile health (mHealth) apps. All these domains, except for healthcare literacy, were raised as important by the stakeholders. Stakeholders also discussed language and communication as one domain, focusing on how limited English proficiency affects a patient’s ability to communicate their needs. Finally, stakeholders also highlighted Social Support as an important domain not widely discussed in the literature.

### Step 3: Identify and use multiple inputs

The third step of the Transcreation Framework is to review the scientific evidence and patient feedback to inform the final design of the intervention. Here, we do this by using two different types of inputs: scientific evidence and input from individuals in the community, in the form of semi-structured interviews. The English-language version of MyHealthyPregnancy is an evidence-based app, designed to assess and communicate pregnancy risks related to preterm birth [16, 20, 21]. Here, we use the baseline content of the English-language version of the MyHealthyPregnancy app, as one input, and conduct literature-informed semi-structured interviews with Spanish-speaking Latino peripartum individuals, as another input, to identify the way that this population approaches prenatal care and their specific needs.

## Patient interviews

We conducted qualitative semi-structured interviews with peripartum Spanish-speaking Latino individuals to characterize their experience related to the main domains of disparity identified in the literature and clarified by the stakeholder interviews (see Table 1). The semi-structured interview instrument included open-ended questions about the domains identified (e.g., “Can you tell us about your worries related to your ability to communicate with your provider?”—language and communication), along with additional open-ended questions about the participant's pregnancy experience to identify any other domains (e.g., “What are some things that are done differently around pregnancy here in the U.S. in contrast to where you or your family came from?”). Additional topics covered in the interviews included participants’ preferred sources of information (family, friends, social media, etc.), barriers to care (transportation, childcare, scheduling) acculturation, medicinal herbs used, the importance of family traditions, and trust in the healthcare system, among others.

**Table 1:** Domains and intervention approaches from stakeholder feedback

Domains discussed	Exemplar quotes	mHealth implementation	
Language and Communication	*"[Patients] want to speak Spanish when they feel vulnerable."* *"Find ways to reduce small language barriers, such as hospital menus, [pregnant individuals] have no information on when to order food, *etc*.—small barriers that exist because of lack of information, caused by lack of language."*	Apps in the patient’s native language Access to and information on translation services Share information in the patient’s native language on what is going to happen during care and delivery	
Financial Concerns	*"It is common for [pregnant individuals] to have to deal with payment problems because most do not have insurance. [We] have to teach them to understand their body signals so that they go to the emergency room when they are about to give birth. At that time, they could apply for emergency insurance."* *"The issue of payment creates stress, that they need to communicate [in English] with the hospital social worker."*	Provide information on navigating health-related financial considerations in the U.S Provide access to information on Federally Qualified Health Centers Offer postpartum services that are accessible without cost	
Social Support	*"Women with low education and socioeconomic status. Commonly, they do not have anyone who supports them. For example, their mothers had no resources or could not come due to immigration permits."* *"They have to limit themselves to a very small circle; they do not have the aunt, the grandmother, the neighbor, in general, the extended network that generally supports Latino women during pregnancy – [they feel that] nothing is not going to happen to me because there are individuals who will take care of me."*	Community support through forums connecting Spanish-speaking pregnant individuals Support programs for doula services that are fluent in the patient’s native language	
Immigration Status	*“Migration problems – they are scared of even leaving the house.”* *“[Pregnant individuals] are worried about telling [healthcare facilities] information and that they may contact their place of employment which could jeopardize their jobs.”*	Access to information on how immigration status will influence (or not influence) their access to care	
Cultural Differences	*“Pregnancy is traditionally more social and managed among the family.”* *“Less-educated women may be focused on using [traditional tea] and other things, natural care, generally this is not communicated to the providers.”*	Acknowledge the importance of family as a source of support and provide ways to enhance links to a community of other Latino individuals Provide information on traditional medicine and how to discuss this topic with providers Offer nutritional advice based on staples of Latino diets	
Healthcare Navigation	*“In Latin America, healthcare is not a product – it is a public service, and the relationship is totally different. They don’t see themselves as customers in the healthcare system.”* *“Mothers must be helped to have the confidence to communicate their problems with nurses, doctors, police (in cases of domestic abuse) – Give them the confidence to communicate with resources themselves.”*	Educate providers on the different doctor-patient relationships in Latino cultures Educate users about their ability to ask questions and participate in their own healthcare decisions	
Location of Services	*“Women will travel if you offer a valuable service… you need to gain trust, once trust is gained you need to give them the tools to overcome a barrier (for example transportation), and then they are highly motivated to go to prenatal classes where they get support.”* *“There is a limited number of organizations offering classes and information, especially in Spanish and in a culturally sensitive way, but the main problem is insurance.”*	Offer spaces for community organizations to offer classes, collaborate, and form a network to support Latino pregnant individuals	

### Participants

We recruited 15 peripartum individuals for the semi-structured interviews, with 14 completing the entire process (one participant withdrew after enrolling due to a scheduling conflict). All interviewees were recruited using community-based strategies, including posting flyers in Federally Qualified Health Centers visited by Spanish-speaking Latino populations, Latino social media groups, and snowball sampling from recruited participants. To participate, individuals had to (1) be 18 years or older, (2) speak Spanish as their primary language, and (3) be pregnant or have given birth within 6 months of the interview. Interviews were conducted in person or by telephone between June and August 2019. For their participation, interviewees were compensated USD 50 for approximately 1 h of their time. All participants had access to a smartphone.

### Interview procedures

The semi-structured interviews started with open-ended questions formulated to suggest potentially relevant topics but not desired answers. As the interview progressed, questions became increasingly focused on the areas identified by our literature and stakeholder interviews. If necessary, responses were followed up with prompts for clarity (e.g., "How does that work?" "Can you explain what you mean a little more?"), as well as prompts that facilitated discussion of the issues. All the interviews were conducted in Spanish.

### Coding

After an initial round of open coding to identify themes by the primary author, complete thematic analysis coding was conducted by a second Spanish-speaking coder.

## Step 4: Design intervention prototype

After analyzing the interviews, a series of specific themes were identified within the domains outlined by our stakeholders and literature review in Steps 1 and 2. We then outlined modifications and updates to app content to address specific needs reflected in those themes. The section below illustrates how these needs were translated into the Spanish language prototype of the MyHealthyPregnancy app for use by pregnant Spanish-speaking Latino people in this community.

## Results

### Identifying and understanding domains of disparity

Table 1 outlines exemplar quotes from community stakeholders on six of the domains identified in the literature review that they considered most relevant to the local emerging Latino community and a seventh, *social support*, that was independently raised by stakeholders and could be considered relevant in the case of any emerging Latino community.

The self-reported demographics of our convenience sample of 14 Spanish-speaking Latino peripartum women are outlined in Table 2. While these demographics may be more reflective of our local emerging Latino population, they are not representative of all US Spanish-speaking Latino immigrants.

**Table 2:** Self-reported demographics (n = 14)

	n (%)
*Country of origin*	
Mexico	5 (36%)
Colombia	3 (21%)
Argentina	2 (14%)
Ecuador	1 (7%)
El Salvador	1 (7%)
Peru	1 (7%)
Honduras	1 (7%)
*Education level*	
Less than High School	2 (14%)
High School	3 (21%)
Some College	2 (14%)
College	5 (36%)
Postgraduate	2 (14%)
*First pregnancy*	
Yes	8 (57%)
No	6 (43%)
*Have used other pregnancy apps during their current pregnancy*	
Yes	8 (57%)
No	6 (43%)

Within the domains described by stakeholders and informed by our literature review, interviewees detailed nuances based on their firsthand experiences. They also emphasized one final domain, connection to local resources as an important need, while less emphasis was placed on the location of healthcare services.

#### Language and communication

This domain includes themes mentioned when participants discussed their ability to effectively communicate with a healthcare provider that does not speak their native language. In this area, participants emphasized *limited English language proficiency* as a limitation to effective communication with providers during prenatal care visits. Some interviewees felt they could not ask follow-up questions for topics they did not understand. This feeling of an inability to speak up and clarify any concerns or issues with the provider's instructions leads to difficulty following through with provider recommendations.

#### Financial concerns

This domain includes themes related to the ability to access healthcare for pregnant participants and their unborn children due to cost. The possible *cost of services* was reported as a significant source of stress during pregnancy and after the baby was born. All interviewees talked about financial worries related to accessing the healthcare that they needed. Three mentioned that they were unsure about the payment process for delivery services and what steps to take at the time of childbirth.

#### Social support

In this domain, we include themes related to community and family support throughout pregnancy and postpartum. Participants mentioned the emotional toll of not having extended family members available, who were often cited as a great source of support in their native countries. This lack of *community and family support* was referenced as a primary cause of stress. 11 out of 14 interviewees later brought up the importance of family support when they expressed their concerns about whom they could reach out to in an emergency or even at the time of delivery.

#### Immigration status

This domain includes participant discussions of the impact of their immigration status on their ability to access care. Interviewees expressed uncertainty in this domain, saying that they were unsure of how their immigration status would affect their pregnancy care and lacked *awareness of rights* when accessing prenatal care. Often these interviewees were fearful of engaging with the healthcare system. Still, most of the interviewees who had immigration concerns decided to engage with medical care despite these fears.

#### Cultural differences

This domain refers to any discussion of traditions in the participant’s family/culture around pregnancy or childbirth. Traditional medicine was discussed as part of cultural traditions but not a central component of their care. When asked about *care alternatives*, eleven participants mentioned using medicinal teas, such as valerian tea or anise star tea, to address joint discomfort. A few also noted that while they might consume the tea occasionally, they did not feel like it was necessary to talk to their provider about it and considered these drinks harmless.

#### Healthcare navigation

This domain includes themes where the participant discusses their experiences navigating the US healthcare system and comparisons between patient-provider relationships. Interviewees commonly discussed significant differences between *patient-provider relationships* in the U.S. and their home countries. Many talked about having a more amicable relationship with their medical providers in their home countries compared to the U.S., which was seen as more transactional. Some discussed that a higher frequency of ultrasounds was available to them in their home countries, which they found reassuring and a way to connect with their baby. Participants observed that providers in the U.S. tended to come into a room and ask quick questions but were not interested in understanding the patient's feelings or developing a relationship, limiting their *comfort* in asking additional questions. Another issue raised by interviewees in their limitation to access services was navigating the complexities of the U.S. healthcare system due to language issues and the intricacies of health insurance.

#### Connection to local resources

This domain refers to the complexity of accessing and connecting with local community resources that offer services in Spanish. Some participants discussed how difficult it had been to *find community resources* for support as they attempted to navigate their pregnancy. Most had resorted to word-of-mouth as they slowly met more Latino immigrants and wished they had a way to access a comprehensive list of all services. Results from the interviews are summarized in Table 3.

**Table 3:** Patient interview results

Domain discussed	Specific themes identified	Exemplar quote	Finding
Language and communication	*Language proficiency:* Lack of services in patient’s native language *Translation quality:* Poor quality of translation services *Access to information:* Lack of information in the patient’s native language on what to expect	*Sometimes the language. There are some doctors who, when they know that your native language is not English, speak slowly or take more pauses, ask you several times if you understood, and then there are others who don't. It has happened to me that sometimes I leave, not knowing if they really understand what I am trying to say. I believe that language is one of the biggest obstacles. [P008]*	Interviewees feel that they are not getting comprehensive information due to language barriers. Additionally, some had bad experiences requesting translation services
Financial Concerns	*Access:* Limited access to Federally Qualified Health Centers *Cost of services:* Inability to pay out of pocket for prenatal care and delivery services	*For the time I am going to have a baby, I do not have the information, on what hospital or how much it will cost me. [P002]*	Financial issues generate stress and anxiety. Often this is linked to feelings of insecurity due to immigration status
Social Support	*Community support:* Lack of community support	*The postpartum experience, I would have liked it better if it was in Colombia and not here because here, I am alone. There you are already incredibly supported, your family arrives, takes care of the baby so that you rest, the meals are great, they are more suitable for the moment of recovery, and in general they help you a lot. [P006]*	Interviewees expressed feeling that they lack emotional support from family members and the need to have someone that cares for them
Immigration Status	*Awareness of rights:* Lack of access to information on how immigration status will influence access to care	*Yes, thinking that in case of an emergency there will be costs to get treatment because since one does not have any papers, I thank God that I have not gotten seriously ill, I have not needed to go– To go to the hospital with an emergency, but they tell me that it costs because you do not have papers and it costs to be seen. As I say, thank God I have not needed to go to a hospital; I have not gotten sick. [P011]*	Interviewees recognized that they need to seek care eventually but often consider access through the emergency room as their only option
Cultural Differences	*Importance of family:* Currently away from extended family *Care alternatives:* Limited information on potential alternative care *Nutrition:* Nutritional advice based on staples of an American diet	*I have been worried about not having many ultrasounds, here in the United States, only two are done, I did not know if this was normal or not, but doctors have told me that two are enough and necessary unless there are any complications, but I would have liked to see the baby a little more, have many more options to see the ultrasound. [P003]*	There is an understanding of traditional medicine, but it is not necessarily considered an essential part of care. There are different expectations on what prenatal care will involve and how it may differ from care in other countries
Healthcare Navigation	*Patient-provider relationships*: Differences in a doctor-patient relationship compared to what is expected in Latino cultures *Comfort:* A feeling of being unable or uncomfortable asking questions *Decision making:* Limited participation in their own healthcare decisions	*In the beginning, it was pretty difficult [to access prenatal care], at some point, because I did not know that I was pregnant, and I came to find out when I was at three months. I did not know how it was here in the United States; how to get [prenatal] care, how to make an appointment, more than anything they told me they spoke to you in English, I don't know any English. For me, it was pretty difficult in that aspect; that's why I started [prenatal] care when I was 28 weeks pregnant. [P002]*	It was difficult for interviewees to find ways to access the healthcare system
Connection to Local Resources	*Community resources*: Lack of a local network to support Latino individuals	*Yes, yes, I would like, let's say, to be able to expand that network of resources and, above all, that they were Latino. Because we know that this city doesn't have a lot of Latino immigrants and we don't have many services… Yes, we don't have many services specific to Latino people. [P003]*	Interviewees emphasized that a critical step is to have a centralized place to access resources available for Spanish-speaking individuals

### Designing a culturally sensitive app to address disparities

The feedback received from our interviewees highlights the critical importance of language and culture on the effectiveness of mHealth app interventions for pregnant immigrants. Table 4 outlines a series of changes and additions necessary to adapt the MyHealthyPregnancy app to the needs of Spanish-speaking Latino pregnant individuals in an emerging Latino community. It highlights a series of needed topics to minimize some existing health disparities in this emerging Latino community. These include information about how to navigate the healthcare system, local resources that offer Spanish language services, and information about immigrant rights to access care.

**Table 4:** New updates to add to the MyHealthyPregnancy prototype

Domain	Topics	Suggested app content
Language and communication	Services offered in their native language Translation services Information on what to expect is offered in the patient’s native language	Include direct links to access translation services available at different healthcare providers, including ways to schedule them ahead of time Provide in-app information on what to expect during appointments and during labor, including questions to discuss with the provider
Financial concerns	Access to Federally Qualified Health Centers	Include in-app information on how to access care in Federally Qualified Health Centers and how to apply for different types of medical assistance
Social Support	Support programs for services offered in the patient’s native language	Include information on how to access programs with Spanish-speaking doulas Discuss different available services that cater to Spanish speakers in the area
Immigration Status	Access to information on how immigration status will influence access to care	Include in-app information on access to prenatal care and alternatives to emergency room care
Cultural Differences	Acknowledge the importance of family and advice received about traditional care practices Nutritional advice based on staples of Latino diets	Update nutritional information to include staples of Latino/Hispanic diets Provide in-app information on what to expect during an appointment, highlighting possible differences from care in other countries
Healthcare Navigation	Educate providers on the different doctor-patient relationships in Latino cultures Educate users to ask questions and participate in their own healthcare decisions	Present in-app information on the different healthcare resources available and how to access each level of care Offer information on how to facilitate involvement in their healthcare
Connection to Local Resources	Offer spaces for community organizations to collaborate and form a network to support users	Maintain an up-to-date list of resources that have been verified as accessible to pregnant Latino individuals independent of their immigration status and that offer services in the patient’s native language

**Fig. 2:**
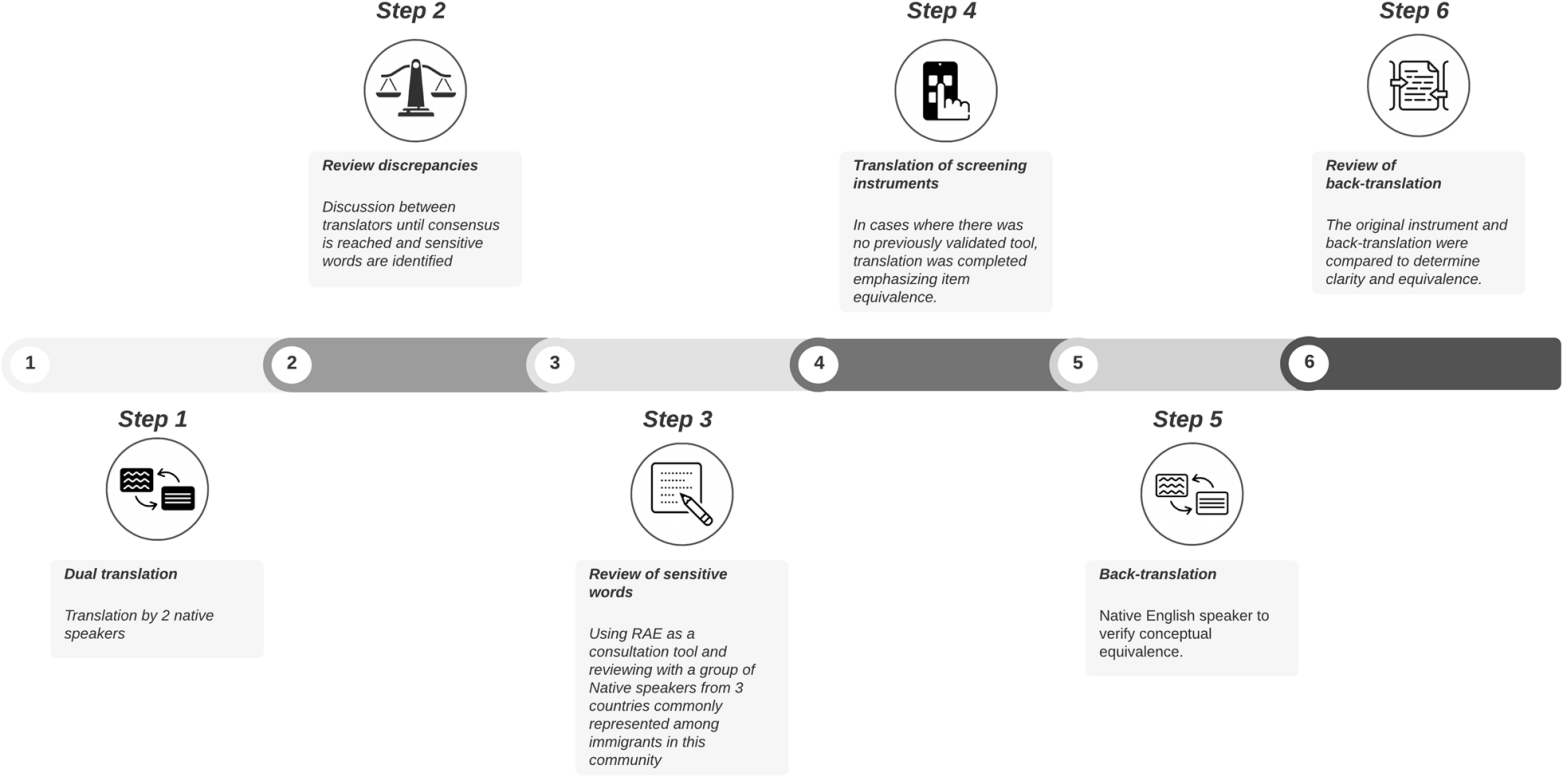
Translation process for mHealth pregnancy app

As part of our efforts to support a Spanish language version of the app, we applied a systematic approach to translating the existing content in the MyHealthyPregnancy app to Spanish, which is outlined in Fig. 2. We identified two goals for this English-to-Spanish language translation. The first was that the translation and validation process would incorporate the nuances in language specific to Spanish-speaking populations from different countries of origin. Our translation team included native speakers from two different Latin American countries, Ecuador and Venezuela, which served as a first pass to identify discrepancies in words. The second goal was that the translation of questionnaires and data collection mechanisms would ensure that the answers were not shaped by how questions were worded [33].

## Discussion

Adequate prenatal care, initiated within the first trimester of pregnancy and increasing in frequency as the delivery date approaches, is associated with a lower risk of prematurity, stillbirth, and neonatal death [34]. Latino pregnant individuals have lower rates of timely initiation of prenatal care, measured by access to care during their first trimester, compared to non-Hispanic whites [35]. For non-US-born Latino pregnant individuals, late prenatal care initiation rates are significantly higher than their US-born counterparts [36–38]. This work adapted an mHealth intervention, the MyHealthyPregnancy app, to acknowledge and offer education on the barriers that may prevent immigrant Spanish-speaking Latino pregnant people from engaging in care.

Our first goal was to understand which existing health disparities for Latino pregnant individuals seeking to access care in an emerging Latino community could be addressed in a mobile health app. We started by identifying possible care access barriers in the context of this local population. Guided by implementation frameworks, we focused on possible barriers that can impact how the clinical encounter is approached and understood. We then talked to Latino peripartum individuals to understand their experiences with those barriers. This formative work was used to determine the necessary app updates.

While not a monolith, our Spanish-speaking Latino interviewees were all immigrants to the US facing barriers to healthcare access, with few available Spanish-speaking services. For each of the barriers discussed with them, we were able to identify specific needs that could be addressed in a pregnancy app. Interviewees commonly discussed how not speaking the provider's language limited their ability to ask questions and understand recommendations. Previous work has identified communication problems between patients receiving prenatal care and providers [36, 37]. Similar to our results, communication problems have also been identified as a factor limiting the value of information from providers [38, 39]. Our interviewees often left appointments with residual questions about follow-up care. mHealth apps that use the patient’s native language can help bridge the communication gap by offering educational content with critical physician-approved information in a proper context to address existing lingering questions and aid in interpreting provider instructions. This can facilitate a clear understanding of medical recommendations as well as equip patients with the knowledge to ask questions and request translation services if necessary.

Interviews with patients were analyzed to identify content requirements to adapt the application. These content requirements include patient educational content that could support prenatal care goals. Existing content was translated through a systematic process that considers qualitative feedback from target audiences. However, this study had certain limitations. We focused on the population of a specific emerging Latino community. While we advocate for a process that is highly tailored to the specific needs of a community, we recognize that the results may not be generalizable to the broader population of pregnant Spanish-speaking Latino individuals in the United States. We also used a convenience sample approach focused on targeting pregnant individuals who were willing to talk to a researcher. It is reasonable to expect that pregnant people with uncertain immigration status may be less willing to engage in research and, as such, our results (and prototype app) may not reflect their unique health care access needs.

While existing methods have attempted to address comprehension issues that result from language translations of validated scales or questionnaires [40, 41], contextual discrepancies in terminology can still be produced by these translation approaches. One example relevant to our pregnancy app intervention relates to the Edinburgh Postnatal Depression Scale (EPDS) screening tool that we use in the app to routinely track depression risk. In the official Spanish language version of the EPDS [42], the word *desgraciada* is used as a translation of the English-language word “miserable.” However, *desgraciada* conveys different meanings in different Spanish dialects (e.g. unlucky, unpleasant, disagreeable, or despicable), altering the interpretation of this question depending on the country of origin of the Spanish speaker [43]. By following recommendations from implementation science and involving native speakers who were the end-users of our product to supplement our translation, we describe a method that can address the translation needs of a Latino community with a focus on how to better support the clinical encounter. This systematic method, which involves final users, also facilitates the implementation of best practices outlined in the previous eHealth literature, including respecting the cultural characteristics of present and future users and respecting the level of literacy of the target population [44].

## Conclusion

Here, we draw on implementation science frameworks to adapt an existing mHealth pregnancy app to the needs of a community of Spanish-speaking Latino individuals. When expanding healthcare access to historically and systematically under-resourced populations, particularly to individuals who speak a primary language that differs from the national norm, there is a need for a comprehensive approach that does not exacerbate existing disparities. Our findings show that there are several instances where a direct translation approach, predicated on a positive patient-provider relationship, would fail to address the healthcare needs of the members of our emerging Latino community. This study focused on a subset of patients from a single emerging Latino community and applied a methodology to modify a specific mHealth pregnancy app. It also focused on a clinical event – pregnancy – for which there are commonly routine touchpoints with the healthcare system. However, the methodological approach to culturally sensitive translation could be expanded to tailor other healthcare tools and processes, including those with limited patient-provider interactions, such as discharge paperwork or medication information leaflets. Similarly, this overarching approach could be used to support ongoing conditions where cultural and language differences may impact how care is managed outside the healthcare system, such as at-home management of chronic conditions or elderly care.

## Data Availability

The data that support the findings of this study are not openly available due to the risks in identifying participants as true anonymization would be difficult to guarantee, but subsets of the data are available from the corresponding author, AC, upon reasonable request.
